# Ipsilateral transfer of motor skill from lower to upper limb in healthy adults: A randomized controlled trial

**DOI:** 10.1371/journal.pone.0303459

**Published:** 2024-05-20

**Authors:** Noa Efrat Sherman, Orit Elion, Zvi Kozol, Moshe Einat, Silvi Frenkel-Toledo

**Affiliations:** 1 Department of Physical Therapy, School of Health Sciences, Ariel University, Ariel, Israel; 2 Department of Electrical and Electronic Engineering, Ariel University, Ariel, Israel; 3 Department of Neurological Rehabilitation, Loewenstein Medical Rehabilitation Center, Raanana, Israel; Institute of Psychology Chinese Academy of Sciences, CHINA

## Abstract

**Background and purpose:**

Whereas motor skills of the untrained upper limb (UL) can improve following practice with the other UL, it has yet to be determined if an UL motor skill can improve following practice of that skill with the lower limb (LL).

**Methods:**

Forty-five healthy subjects randomly participated in a 10-minute single-session intervention of (1) practicing 50 reaching movement (RM) sequences with the non-dominant left LL toward light switches (LL group); or (2) observing the identical 50 light switches sequences (Switches Observation (SO) group); or (3) observing nature films (Nature Observation (NO) group). RM sequence performance with the left UL toward the light switches was tested before and immediately after the intervention and retested after 24 h.

**Results:**

Reaching response time improved in the LL group more than in the SO and NO groups in the posttest (pBonferroni = 0.038 and pBonferroni < 0.001, respectively), and improved in the LL group more than in the NO group in the retest (pBonferroni = 0.004). Percentage of fails did not differ between groups across the timepoints.

**Conclusions:**

It appears that the actual practice of the RM sequence skill with the UL together with the cognitive element embedded in the observation of the RM sequences contributes to ipsilateral transfer from LL to UL.

## 1. Introduction

The ability to acquire new motor skills is essential for interacting with the environment throughout the life span, including in rehabilitation following injury to the central nervous system. Skilled performance becomes more specific when more practice is afforded [1–4]. However, under certain conditions, some practiced skills may be intermanually transferred (intermanual transfer) to the performance of different skills or to other effectors (e.g., the contralateral limb) [5].

Intermanual transfer is related to the constraints and phases of motor skills acquisition [4,6,7]. Generally, there are two phases: an initial fast phase that relates to within-session gains, followed by a slow evolving between-session gains phase [6,8–11]. The between-session gains lead to an enduring and robust memory of the skill [6]. Once the learned skill has become specific in long-term memory, transfer of the gains from the learned task to a novel task is less likely [4,6,12].

Evidence exists for the intermanual transfer of strength [13–16] and motor skill [17–22]. A meta-analysis showed that the unilateral training of the UL or LL resulted in a 15%-29% increase in the strength of the homologous muscles in the contralateral limb of young and healthy adults, as well as adults with orthopedic or neurological impairments [14]. In addition, unilateral training of the UL resulted in improved or maintained strength of the contralateral, immobilized UL [23,24]. With regard to motor skill, the reaction time of the finger sequence was transferred from the trained effector to the contralateral untrained effector [21,22], and the speed component rather than the accuracy component of a star tracing task was intermanually transferred [22].

In contrast to the evidence concerning the intermanual transfer of the UL [13–22], very few studies have investigated ipsilateral transfer within the same UL (intramanual transfer) [18,25] and between limbs [26–28]. For example, transfer of a motor skill, in which the participants had to track the head of a snake (2D virtual “moving snake” task), has been found from the shoulder to the finger [18]. After shoulder training, the accuracy index (the mean spatial error) of the finger improved. In a study of ipsilateral transfer from the LL to the UL, an increase in the 1 repetition maximum of the ipsilateral biceps brachi was found following a ten-week leg press resistance training program of the LL [26]. The increase of UL strength was greater after the training of the UL biceps muscle, which was immediately followed by leg press, as compared to training of only the UL biceps [28]. In addition, leg press training of the dominant LL in children resulted in both ipsilateral and contralateral increases in elbow flexor strength and grip force [27]. It has been suggested that ipsilateral transfer between non-homologous effectors requires the intra-hemispheric transfer of information [18].

Whereas the abovementioned studies describe training that triggered the intermanual transfer of strength and motor skills [13–24] and the ipsilateral transfer of strength from the LL to the UL [26–28], to the best of our knowledge, no current data exists regarding the ipsilateral transfer of motor skills from the LL to the UL and vice versa. This study is the first attempt to determine whether there is an ipsilateral transfer of a motor skill from the LL to the UL, which can potentially provide additional insights about transfer principles and possible clinical applications. Specifically, we investigated whether practicing reaching movement (RM) sequences with the LL toward light switches can be transferred to the UL in healthy adults. The process of sequence learning involves two separate components: first, acquiring the arrangement of elements in the sequence, and second, being able to execute the sequence, thereby merging the elements into a single skilled action. As cognition plays a role in motor learning, particularly when it comes to choosing actions at the correct time and in the correct sequence [29], we compared the RM sequences practice to merely observing the same sequences of the light switches. In this manner, we sought to compare the contribution of the cognitive aspect (which is related to the memory of the sequence) vs. the combined cognitive and motor aspects to the ipsilateral transfer of RM sequences. We hypothesized that practicing RM sequences with the LL would improve the performance of RM sequences with the ipsilateral UL compared to merely observing the same sequences of the light switches or observing nature films.

## 2. Method

### 2.1. Study design

This was a single-blind, parallel, randomized, controlled study. Data were collected in a brain and motor behavior laboratory based at Ariel University, Israel. Subjects were randomly assigned with a 1:1:1 ratio, using a random number generator in WINPEPI, to one of three groups: (1) practice of RM sequence with the LL toward light switches (LL group); (2) observation of sequence of light switches (Switches Observation (SO) group); and (3) observation of nature films (Nature Observation (NO) group). All participants were blinded to group allocation. Research assistants who administered the intervention and measured the outcomes received allocation information via coded email from the researcher SFT. Blinding of group allocation was maintained during the data analysis. The trial was retrospectively registered at the ClinicalTrials.gov registry on 30/07/2023 with trial registration number NCT05988775. All methods were performed in accordance with the relevant guidelines and regulations.

### 2.2. Participants

**Fig 1:**
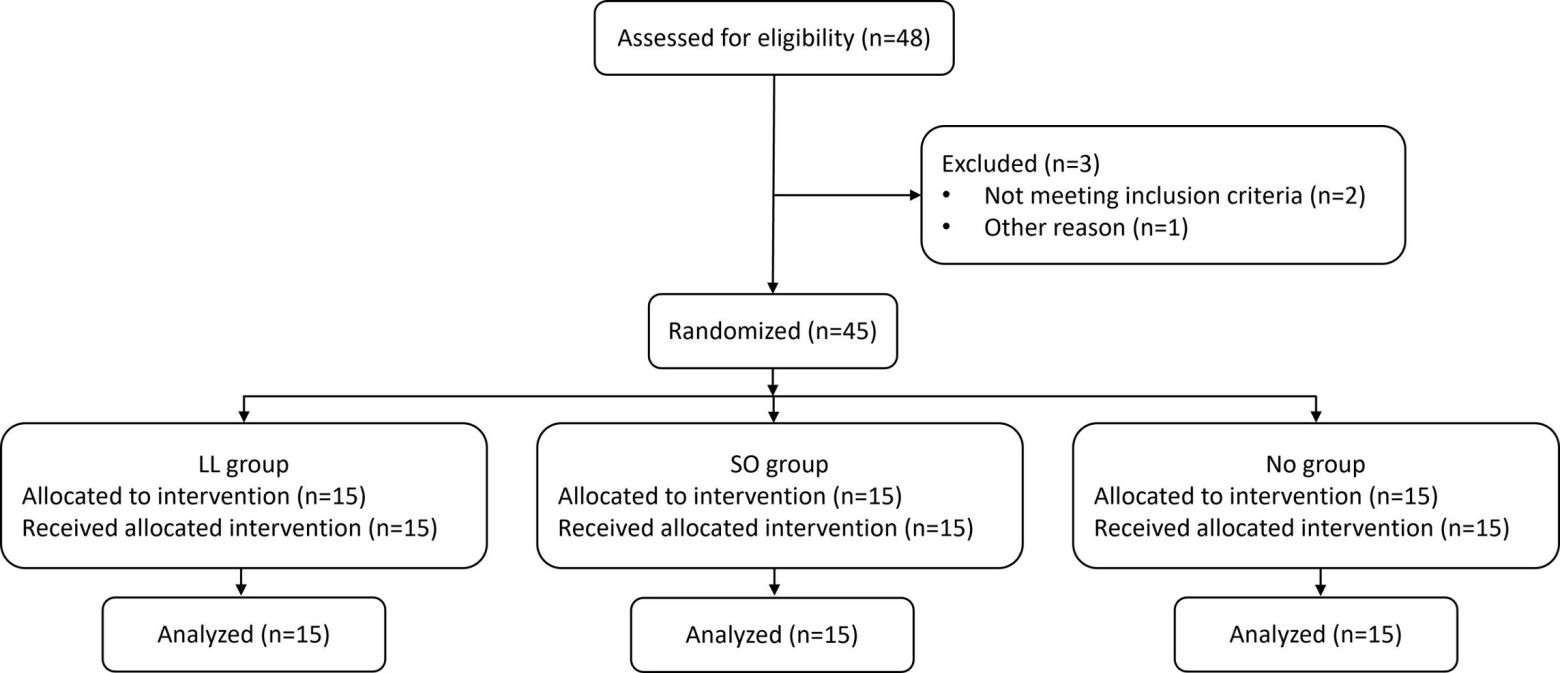
Trial flowchart. LL group = lower limb group that practiced the RM sequence with the LL toward light switches; SO group = switches observation group that observed the sequence of light switches; NO group = nature observation group that observed nature films.

The sample size for this study was determined based on a power analysis calculation that was conducted using G*Power version 3.1.9.7. Power analysis yielded a total sample size of 45 individuals (15 individuals per group) for detecting significant interaction with an assumed effect size of 0.25 and a power of 90%. Forty-five subjects (23 women; aged 25 ± 3 years) participated in the study between May 9th, 2022 to July 26th, 2022. Inclusion criteria included being aged between 20 and 35, right-hand dominance and self-report regarding being healthy. Exclusion criteria included having musculoskeletal or neurological deficits interfering with task performance (proper UL and LL reaching performance). The study was approved by the Ethics Committee of Ariel University (approval number: AU-HEA-OE-20210610). Written informed consent was obtained from all participants involved in the study. The Consolidated Standards of Reporting Trials (CONSORT) recommendations (CONSORT Checklist) are followed in our study; a CONSORT flow diagram is shown in Fig 1.

### 2.3. Motor task

Subjects took part in two sessions. The initial session involved familiarization practice of the motor task, a pretest, a single session intervention (based on group randomization), and a posttest. The second session comprised a retest conducted 24 hours after the training. The familiarization practice and tests were conducted with the UL, and the single session intervention was carried out with the LL.

**Fig 2:**
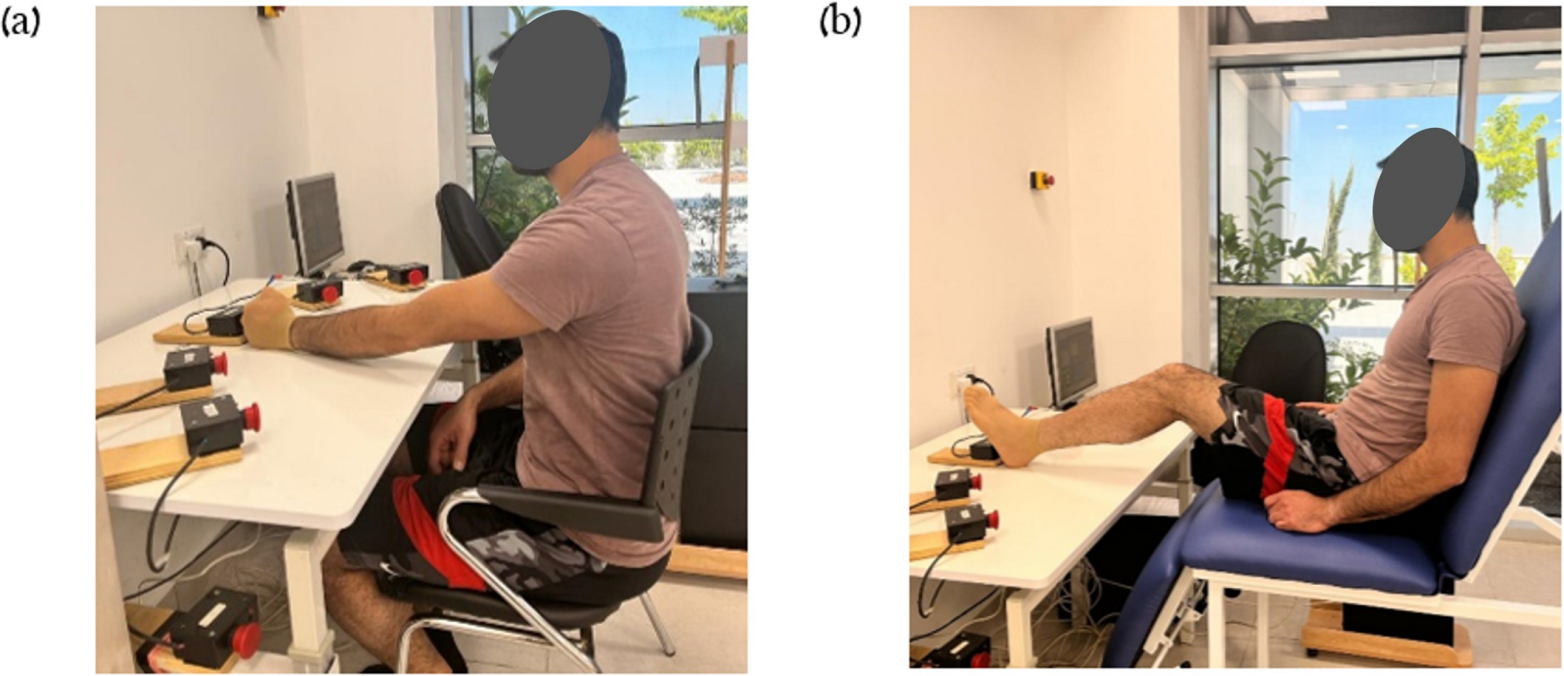
General setup. (a) performance with the upper limb. (b) performance with the lower limb. To evaluate upper limb performance, the subjects performed reaching movement sequences with the left upper limb toward the units. During the single session intervention, the subjects performed reaching movement sequences with the left leg towards the units.

The recording device used in tests (pretest, posttest, and retest) consisted of a custom-made testing apparatus set up on an adjustable height rectangular table with a smooth laminated tabletop of 105 cm by 80 cm. Five switch-led units of 5 cm by 8 cm by 5 cm were connected to the tabletop in a half circle with a radius of 38 cm, numbered from 1 to 5. Each unit was comprised of a large push-button switch and a red light-emitting diode (LED). A computer, interfaced with a LabVIEW software data acquisition card, operated the system. The initiation of a particular unit’s LED served as a signal to reach towards that unit and press the push-button switch. Deactivating the unit involved reaching for its switch, and the response time between the activation and deactivation of the LED was recorded. Reaching toward the switch of an activated unit deactivated it, and the response time between the activated and deactivated LED was recorded. A detailed description of the task and the apparatus is provided in a previous study [30]. To evaluate UL performance, the subjects sat on a chair with sturdy back support, placed in front of a table, ensuring their hips and knees flexed at a 90-degree angle. Participants initially positioned their left fist at the table’s edge in front of their chest (aligned parallel to switch 3). This placement allowed them to extend and touch switch 3 with their third right metacarpal (Fig 2A). The individual in this manuscript has given written informed consent (as outlined in PLOS consent form) to publish these case details (in Fig 2).

Participants engaged in a familiarization practice involving 15 RM in three sequences 1-4-3-5-4-2. This practice entailed reaching with their left UL towards the activated unit, as quickly as possible, and returning the UL to the starting position. This process continued until the next unit was activated, with an activation duration and delay of 1 s. The subjects were instructed to reach as quickly and accurately as possible with the left UL from the starting position to the light switch, press it, and then return to the starting position. Throughout this action, they were instructed to ensure that their fist remained in contact with the table. All groups were informed about the sequence 1-4-3-5-4-2 in the familiarization practice. In each pretest, posttest, and retest, the subjects also performed RMs with their left UL toward units that were activated in the identical sequence 1-4-3-5-4-2, maintaining an activation duration and delay of 1 s. The subjects executed two 5 sequences (i.e., 60 RMs/trials); five sequences constituted a block (i.e., two blocks). If the subject failed to reach toward the activated unit and touched the unit-related switch within 1 s, the trial was deemed a ‘‘fail” and was excluded from the averaged response time. After each block, the subjects rested for 30 s. Outcome measures were made by averaging the response time of all the RMs during the sequences (ms) (primary outcome measure), and the percent of fails was calculated for each block as (number of fails/30 trials)*100 (secondary outcome measure). Improved motor performance was indicated by a shorter response time and fewer failures.

### 2.4. Procedure of single session intervention

In each of the LL, SO and NO groups, a 10-minute single-session intervention was conducted. Subjects of the LL group sat on a custom-designed plinth with a solid back support in front of the apparatus at the same height as the tabletop; hence, they could perform the RM sequence with the leg. At the starting position, the heel was placed on the edge of the table in front of switch 3, so while the left heel touched switch 3, the knee reached 30° of flexion (Fig 2B). The initial testing position of the SO and NO groups during the intervention was sitting on a chair with solid back support, hips and knees flexed 90°, in front of the apparatus used for the tests. The LL group was instructed to reach with the left LL from the starting position as fast and accurate as possible to the light switch, press it, and return to the starting position, while the heel must remain in contact with the table. The subjects performed RMs toward the units that were activated in the same order as the tested sequence 1-4-3-5-4-2, with an activation duration and delay of 1 s. The practice included 10 blocks, each consisting of 30 RMs with a 30 s pause after each block. They were informed about the sequence. The SO group was instructed to observe the light switches while avoiding moving. The subjects observed RMs toward the units that were activated in the practiced sequence 1-4-3-5-4-2, also with an activation duration and delay of 1 s and 30 s pause after each block. Top of Form

They were informed about the sequence. The NO group was instructed to observe a video clip while avoiding moving. The video clip consisted of a 10 min nature movie in cycles of one-minute observation and pausing 30 s, equivalent to the timing of RMs performed by groups LL and SO.

### 2.5. Statistical analysis

Age and sex were compared between groups (LL, SO, NO) using one-way ANOVA and chi-squared tests, respectively. Normal distribution was found for response time and not for percent of fails. Differences between groups in the pretest, regarding response time and percent of fails, were investigated using one-way ANOVA and Kruskal–Wallis with Bonferroni correction for multiple comparisons, respectively. The effects of practice and time on the response time were investigated using a mixed-design ANOVA with time (pretest, posttest, retest) as the within-subject factor and group (LL, SO, NO) as the between-subject factor with Bonferroni correction for multiple comparisons. Due to the non-normal distribution of the percent of fails, subtraction values between each two tests (pretest-posttest, pretest-retest, posttest-retest) were compared between groups using Kruskal-Wallis test with Bonferroni correction for multiple comparisons. All tests were performed using SPSS (version 26.0) with initial significance levels of p < 0.05.

## 3. Results

Forty-eight participants underwent the pre-enrollment screening evaluation. Of those, two did not meet the inclusion criteria and one had technical problems with the device. Age (LL group: 24.9 ± 1.8 years; SO group: 24.9 ± 3.2 years; NO group: 25.0 ± 2.7 years) and sex (LL group: eight women; SO group: seven women; NO group: eight women) did not differ between groups (p > 0.951, for all). Individual data are displayed in S1 Table.

### 3.1. Motor sequence learning task

Mean values of response time (ms) and percent of fails by group and time are shown in Table 1. Response time and percent of fails did not show significant differences between groups in the pretest (p = 0.840, p = 0.903, respectively).

**Table 1:** Means, standard deviations and confidence intervals of response time and percent of fails for groups in time points.

Variable	LL group (n = 15)			SO group (n = 15)			NO group (n = 15)		
	Pretest	Posttest	Retest	Pretest	Posttest	Retest	Pretest	Posttest	Retest
Response time (ms)	531.09± 128.63 [459.86–602.33]	284.24± 108.61 [224.08–344.39]	304.21± 121.37 [237–371.43]	551.30± 140.11 [473.70–628.89]	407.29± 133.69 [333.25–481.32]	400.43± 142.81 [321.34–479.52]	559.71± 139.69 [482.35–507.21]	482.91± 143.07 [403.67–562.14]	475.14± 141.84 [396.56–553.69]
Fails (%)	2.11 ± 2.55 [0.69–3.52]	3.11 ± 2.50 [1.72–4.50]	2 ± 2.53 [0.59–3.40]	3 ± 3.89 [0.84–5.15]	1.22 ± 1.93 [0.14–2.29]	2.33 ± 2.58 [0.90–3.76]	2.22 ± 2.72 [0.71–3.72]	1.55 ± 1.72 [0.60–2.50]	1 ± 1.22 [0.32–1.68]

ms = milliseconds. LL group = lower limb group which practiced reaching movements sequence with the LL towards light switches; SO group = switches observation group which observed the sequence of light switches; NO group = nature observation group which observed nature films.

Effects on response time (ms):

A main effect of Time (F(2,84) = 66.649; p < 0.001; partial η2 = 0.613; observed power = 1.00) showed that, overall, response time was shorter in the posttest (391.48 ± 151.03 ms; pBonferroni = 0.001) and retest (393.26 ± 150.28 ms; pBonferroni = 0.001) than in the pretest (547.37 ± 133.67 ms; pBonferroni = 1.000). A main effect of group (F(2,42) = 4.686; p = 0.015; partial η2 = 0.182; observed power = 1.00) showed that, overall time points, response time was shorter in the LL group (373.18 ± 137.12 ms) than in the NO group (505.92 ± 46.74 ms).

**Fig 3:**
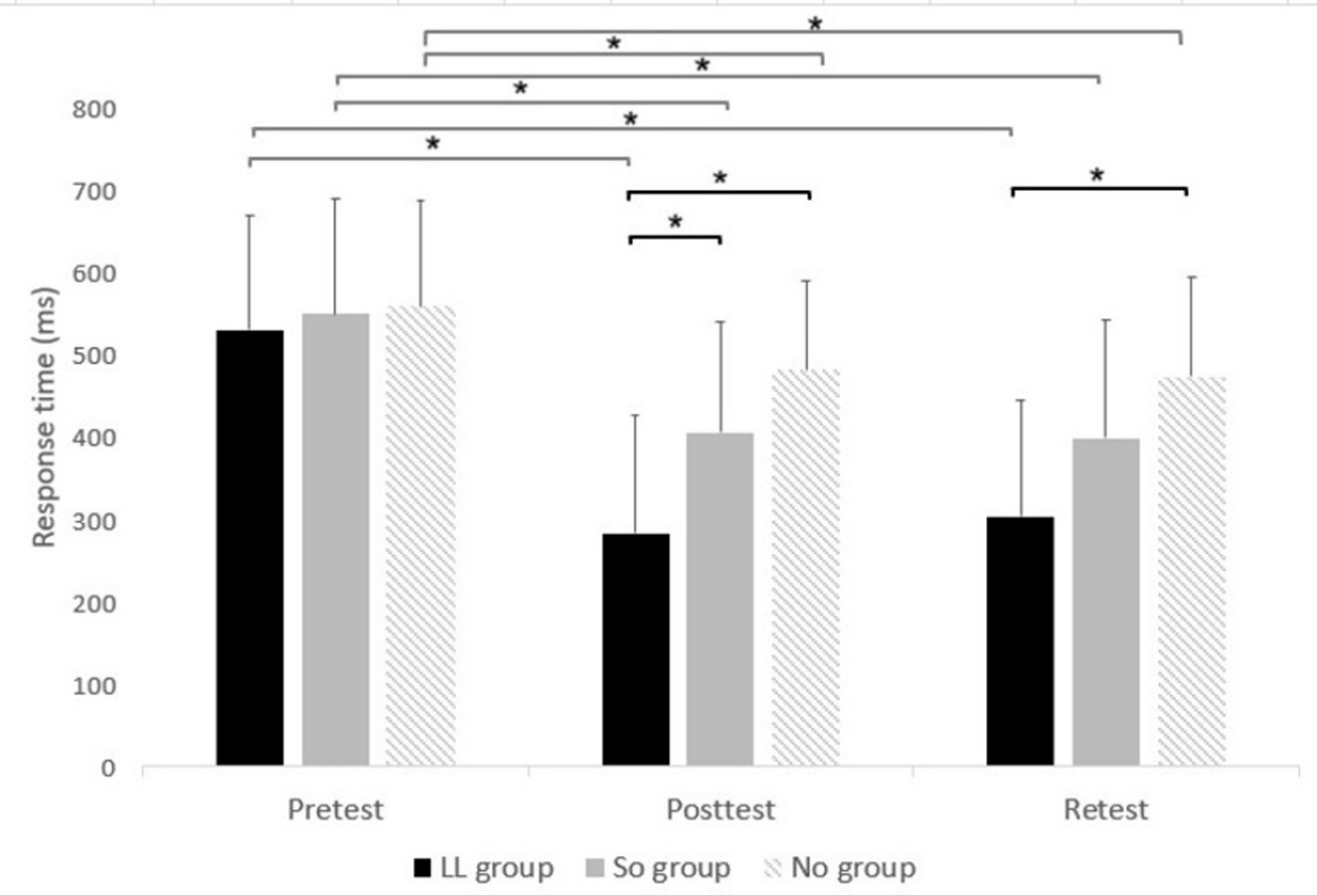
Response time (ms) of reaching movements (RMs) during all sequences in each group at the different time points. Asterisks denote a significant difference (pBonferroni < 0.05). LL group = lower limb group that practiced the RM sequence with the LL toward light switches; SO group = switches observation group that observed the sequence of light switches; NO group = nature observation group that observed nature films.

However, there was an interaction of Group x Time (F(4,84) = 5.851, pBonferroni < 0.001; partial η2 = 0.218; observed power = 0.945). In the posttest, response time differed between groups (F(2,44) = 9.024; p < 0.001) such that it was significantly shorter in the LL group (284.24 ± 108.61 ms) than in the SO group (407.29 ± 133.69 ms; pBonferroni = 0.038) and NO group (482.91 ± 143.07 ms; pBonferroni < 0.001). In the retest, response time differed between groups (F(2,44) = 5.98; p = 0.005) such that it was significantly shorter in the LL group (304.21 ± 121.37 ms) than in the NO group (475.14 ± 141.84 ms; pBonferroni = 0.004). In addition, in each group, response time decreased significantly in the posttest and retest compared to the pretest (pBonferroni < 0.001, for all; Fig 3). No other significant effects were observed.

Effects on percent of fails (%):

Subtraction values did not differ between the groups (p≥ 0.065, for all).

## 4. Discussion

To the best of our knowledge, this is the first study that evaluated whether there is an ipsilateral transfer of a motor skill from the LL to the UL. We found that in the posttest, the response time of RM sequences of the UL was significantly faster (shorter) in the group that practiced the RM sequence with the LL (LL group) as compared to the group that observed a sequence of light switches (SO group) and the group that observed nature films (NO group), whereas it did not differ in the pretest between these groups. In the retest, the response time of RM sequences of the UL was significantly faster in the LL group compared to the NO group. In addition, in each group, response time improved significantly in posttest and retest compared to the pretest. The percent of fails did not differ between groups at the different time points.

Our finding that, in the posttest, the response time of RM sequences of the LL was significantly faster in the LL group as compared to both the SO and NO groups is in line with our hypothesis that LL practice would improve UL performance compared to merely observing the same sequences of the light switches or observing nature films. This finding regarding the ipsilateral transfer of performance from LL to UL supports the findings of the few previous studies that investigated ipsilateral transfer of strength from the LL to the UL in healthy adults and youth [26,27,28], and the ipsilateral transfer of a motor skill (2D virtual “moving snake” task and star-line drawing task [29]) between proximal and distal effectors within the UL [18,29].

The finding related to the ipsilateral transfer of a motor skill from the LL to the UL complements previous findings regarding the ipsilateral transfer of strength due to behavioral and neural evidence for dissociation between strength and motor skill [31–33]. From the behavioral point of view, for example, a finger flexor control abnormality, which was not attributable to weakness, was demonstrated in poststroke patients [32]. They showed more enslaving of passive fingers for any submaximal voluntary force, that is, even when normalizing for their weakness, they still had markedly less control. From a neural perspective, experimental evidence shows that the reticulospinal tract may be particularly important for generating higher force muscle contractions [33].

Our data are also in agreement with the generalized motor program theory. This theory considers motor learning as the generation of an abstract memory structure (i.e., a motor program), which enables a performer to adapt a learned skill to altering environmental requirements [5]. This central motor representation is hypothesized to be independent of the effector used, as reflected in inter- and intramanual transfer. With regard to intermanual transfer, a key role is probably played by the corpus callosum, the largest white matter tract connecting the two cerebral hemispheres (but see also [34]). However, an ipsilateral transfer between limbs on the same side or within the same limb may require intrahemispheric transmission of information. Alternatively, ipsilateral transfer can be explained by shared representation in the rolandic motor association (RMA) region, a motor association area, which was recently found in the depths of the central sulcus [35,36]. The RMA was found to be electrophysiologically active during tongue, hand or foot movements [36]. The authors suggested that because the RMA is not plainly related to any single movement function, it is probably an association area that helps coordinate different effectors of movement.

The task of RM sequence performance includes motor (reaching performance) and cognitive aspects (sequence of light switches). Actually, any real-world motor task necessarily entails both cognitive and movement components [29]. The LL and SO groups were explicitly instructed about the sequence order at the beginning of the task in order to focus on examining improvements in the motor performance of the sequence, rather than on the learning of the sequence order itself. In the single session intervention, the LL group was instructed to reach with the LL from the starting position as quickly and accurately as possible to the light switch, press it, and return to the starting position, whereas the SO group was instructed to observe the light switches while avoiding moving. The cognitive aspect was also related to the repeated exposure to the light switches of the sequence during the single session intervention, which included 50 sequences (in both the LL and SO groups) as an activation (illumination) of a specific unit LED was a cue for the subjects to reach toward that unit and press the push-button switch. This cognitive aspect of the task could have also led to the improved response time of the RM sequences of the UL. By comparing the LL and SO groups, we sought to disentangle the cognitive aspect (SO group) from the inherent combination of cognitive and motor aspects in the current task (LL group). The findings that, in the posttest, the response time of the UL RM sequences was better in the LL group than in the SO group, and that, in the posttest, response time of the UL RM sequences was not better in the SO group than in the NO group (while pretest values were similar in all the groups) suggest that just practicing the cognitive aspect of the task (being exposed to the light switches of the sequence) was not sufficient for triggering ipsilateral transfer from the LL to the UL. Therefore, it seems that the combined practice of the motor and cognitive aspects was required to trigger ipsilateral transfer from the LL to the UL.

Indeed, there is evidence that intermanual transfer can be facilitated by a cognitive strategy [17,37–40]. Explicit (cognitive) processes were found to be primarily responsible for intermanual transfer of a visuomotor adaptation task when participants adapted to a large visuomotor distortion of which they were aware [17]. Elements of the task environment, such as the type of visual feedback available, can also alter the balance between cognitive strategies and motor adaptation and affect intermanual transfer [39]. Intermanual transfer was facilitated in an endpoint feedback condition that consisted of cognitive strategy. The participants isometrically exerted force on a handle to adjust the height of the visual bar on the screen to a target level. Visual feedback was continuously provided for one group, while only the endpoint of the force trajectory was presented to another group. It was suggested that restricted visual feedback to the endpoint relied heavily on a cognitive strategy to solve the task because reaction times increased in that task. Intermanual transfer was facilitated in the endpoint feedback condition, suggesting that effector-independent learning was facilitated by a cognitive strategy [39]. Despite the differences of experimental design and tasks in the above-mentioned studies [17,37–40], the cognitive aspect, which is inherent in the task, improved the intermanual transfer. It should be noted that our study design did not aim to elucidate the respective contributions of the cognitive and motor aspects to ipsilateral transfer.

On the other hand, there is also contradictory evidence that intermanual transfer does not depend on cognitive awareness of visuomotor perturbation [40]. Even informing the participants about the rotation prior to the adaptation session (presumably leading to full awareness) did not lead to increased intermanual transfer compared to adaptation without explanation [41]. In another experiment, in which the degree of awareness of the visuomotor rotation was manipulated by introducing a 22.5° perturbation in either an abrupt single step or gradually in ~ 1° increments every 10 trials, intermanual transfer was similar in both the abrupt and gradual groups, suggesting that awareness of the perturbation has little effect on intermanual transfer [42]. It is possible that these studies on visuomotor adaptation failed to demonstrate the effect of cognitive awareness on transfer because of the small perturbation sizes (32 deg [41] and 22.5° [42]) which probably did not lead to awareness. Even perturbations as large as 40° engaged very little awareness [43]. Awareness was indeed found to depend on perturbation size [43], and the extent of the participants’ awareness of the learned perturbation was directly related to the amount of intermanual transfer [38]. Werner et al. [43], examined interlimb transfer in four conditions in which the rotation size was 30° or 75°, and the rotation was provided either gradually or abruptly. The authors measured indexes of awareness and unawareness separately, and the results indicated that both awareness and transfer were larger in the abrupt 75° condition. It should be noted that the extent of the transfer was found to differ depending on additional factors such as which hand is trained first [44] and the location of the targets in the workspace [45].

The response time of RM sequences improved in all groups in the posttest and retest compared to the pretest but did not improve further from the posttest to the retest, i.e., there was an initial within-session gain but there was no off-line consolidation. This finding in the NO group emphasizes that the number of RM sequences repetitions practiced by the UL during the pretest and posttest was not enough for consolidation of UL response time in the retest. The finding that the response time of RM sequences of the UL was significantly faster in the LL group compared to the SO group in the posttest but not in the retest implies that the ipsilateral transfer of performance from the LL to the UL in the posttest did not fully consolidate to the retest [46]. It is yet to be determined if practicing a larger number of LL repetitions would produce ipsilateral transfer to the UL in the retest as well.

### 4.1. Limitations of the study

First, the experimenter was not blinded to group allocation. It should be noted, however, that the scoring of the motor task was automatically computed by the LabVIEW software. Second, conducting separate measurements for reaction time and movement time could have enhanced the focus on the ipsilateral transfer of the motor performance itself, which is primarily reflected in the movement time.

### 4.2. Conclusions

Our results provide evidence for the ipsilateral transfer of a sequential motor skill from the LL to the UL in healthy adults. These findings pave the way for further studies that can combine behavioral measures with neural measures (using, for example, transcranial magnetic stimulation or electroencephalography) to elucidate the neural mechanism underlying ipsilateral transfer between LL and UL. Ipsilateral transfer of motor skills may have practical implications and consequences for skill development in sports and rehabilitation settings.

## Supporting information

S1 Checklist (DOCX)

S1 Table Individual data in time points. ms = milliseconds. LL group = lower limb group which practiced reaching movements sequence with the LL towards light switches; SO group = switches observation group which observed the sequence of light switches; NO group = nature observation group which observed nature films.
(DOCX)
