# Supplementary material for: Ipsilateral transfer of motor skill from lower to upper limb in healthy adults: A randomized controlled trial
Source: PLoS One. 2024 May 20;19(5):e0303459. doi: 10.1371/journal.pone.0303459 (PMC11104604; doi:10.1371/journal.pone.0303459)
Supplement: S1 Table — ms = milliseconds. LL group = lower limb group which practiced reaching movements sequence with the LL towards light switches; SO group = switches observation group which observed the sequence of light switches; NO group = nature observation group which observed nature films. (DOCX) [file pone.0303459.s002.docx]

S1 Table:

**Title:** **Individual data in time points**

| Subject code | Group | Age | Sex | Pretest-Response time (ms) | Posttest-Response time (ms) | Retest-Response time (ms) | Pretest-Fails (%) | Posttest-Fails (%) | Retest-Fails (%) |
| --- | --- | --- | --- | --- | --- | --- | --- | --- | --- |
| 1 | SO | 25 | 0 | 475.2 | 427.9 | 458.7 | 0.0 | 0.0 | 0.0 |
| 2 | NO | 27 | 0 | 549.6 | 434.4 | 469.7 | 1.7 | 0.0 | 1.7 |
| 3 | NO | 28 | 0 | 544.3 | 501.2 | 528.2 | 1.7 | 0.0 | 1.7 |
| 4 | SO | 26 | 0 | 452.7 | 471.7 | 475.8 | 1.7 | 1.7 | 1.7 |
| 5 | NO | 23 | 1 | 299.5 | 237.4 | 340.8 | 0.0 | 0.0 | 3.3 |
| 6 | NO | 23 | 1 | 673.1 | 662.8 | 706.8 | 0.0 | 1.7 | 1.7 |
| 7 | LL | 24 | 1 | 583.2 | 193.2 | 251.4 | 6.7 | 5.0 | 1.7 |
| 8 | LL | 27 | 1 | 587.7 | 251.9 | 184.3 | 1.7 | 1.7 | 3.3 |
| 9 | LL | 27 | 0 | 556.0 | 346.1 | 437.2 | 6.7 | 6.7 | 1.7 |
| 10 | LL | 25 | 0 | 607.6 | 277.4 | 148.0 | 0.0 | 3.3 | 3.3 |
| 11 | LL | 25 | 1 | 452.2 | 163.8 | 416.3 | 0.0 | 1.7 | 1.7 |
| 12 | SO | 20 | 1 | 482.6 | 405.5 | 263.0 | 8.3 | 6.7 | 1.7 |
| 13 | SO | 22 | 1 | 718.7 | 282.9 | 199.6 | 5.0 | 0.0 | 5.0 |
| 14 | SO | 20 | 1 | 816.8 | 640.6 | 672.3 | 1.7 | 3.3 | 3.3 |
| 15 | LL | 22 | 1 | 683.6 | 382.8 | 379.5 | 1.7 | 0.0 | 8.3 |
| 16 | NO | 24 | 1 | 580.2 | 555.4 | 523.9 | 8.3 | 1.7 | 1.7 |
| 17 | SO | 25 | 0 | 388.0 | 404.6 | 328.2 | 1.7 | 1.7 | 0.0 |
| 18 | LL | 27 | 0 | 232.9 | 161.7 | 190.5 | 0.0 | 3.3 | 0.0 |
| 19 | LL | 25 | 0 | 549.2 | 383.1 | 419.8 | 0.0 | 5.0 | 6.7 |
| 20 | SO | 26 | 1 | 392.4 | 290.4 | 281.5 | 3.3 | 0.0 | 3.3 |
| 21 | NO | 24 | 0 | 736.7 | 703.2 | 657.2 | 1.7 | 1.7 | 0.0 |
| 22 | NO | 24 | 0 | 582.7 | 465.7 | 495.5 | 3.3 | 1.7 | 1.7 |
| 23 | LL | 23 | 1 | 622.2 | 246.3 | 366.2 | 1.7 | 1.7 | 0.0 |
| 24 | LL | 24 | 1 | 249.9 | 183.4 | 148.7 | 6.7 | 1.7 | 1.7 |
| 25 | SO | 24 | 1 | 399.0 | 194.3 | 276.5 | 0.0 | 3.3 | 1.7 |
| 26 | NO | 23 | 0 | 364.1 | 309.6 | 247.1 | 0.0 | 1.7 | 0.0 |
| 27 | LL | 23 | 1 | 614.9 | 314.3 | 311.9 | 3.3 | 3.3 | 0.0 |
| 28 | NO | 26 | 1 | 619.1 | 470.4 | 491.7 | 8.3 | 5.0 | 0.0 |
| 29 | LL | 27 | 1 | 592.7 | 238.3 | 276.5 | 0.0 | 5.0 | 0.0 |
| 30 | SO | 26 | 0 | 594.9 | 477.0 | 507.6 | 0.0 | 0.0 | 1.7 |
| 31 | SO | 25 | 1 | 557.6 | 241.0 | 318.1 | 0.0 | 0.0 | 0.0 |
| 32 | LL | 23 | 0 | 572.9 | 211.3 | 173.9 | 1.7 | 0.0 | 1.7 |
| 33 | NO | 20 | 1 | 674.4 | 638.3 | 549.0 | 3.3 | 3.3 | 0.0 |
| 34 | SO | 25 | 0 | 738.3 | 400.5 | 399.2 | 3.3 | 0.0 | 1.7 |
| 35 | SO | 24 | 0 | 402.5 | 282.8 | 228.8 | 0.0 | 0.0 | 0.0 |
| 36 | SO | 29 | 0 | 688.9 | 644.8 | 603.9 | 13.3 | 0.0 | 8.3 |
| 37 | NO | 24 | 1 | 506.8 | 457.2 | 464.2 | 0.0 | 0.0 | 0.0 |
| 38 | NO | 30 | 0 | 512.0 | 402.4 | 443.3 | 1.7 | 1.7 | 3.3 |
| 39 | SO | 24 | 1 | 556.6 | 515.8 | 518.3 | 0.0 | 0.0 | 0.0 |
| 40 | NO | 25 | 1 | 774.2 | 651.5 | 593.6 | 1.7 | 5.0 | 0.0 |
| 41 | NO | 24 | 1 | 638.0 | 488.7 | 453.8 | 1.7 | 0.0 | 0.0 |
| 42 | LL | 24 | 0 | 514.0 | 343.9 | 317.2 | 1.7 | 0.0 | 0.0 |
| 43 | LL | 28 | 0 | 547.7 | 566.1 | 542.0 | 0.0 | 8.3 | 0.0 |
| 44 | SO | 33 | 0 | 605.5 | 429.6 | 475.0 | 6.7 | 1.7 | 6.7 |
| 45 | NO | 30 | 0 | 341.0 | 265.5 | 162.4 | 0.0 | 0.0 | 0.0 |
